# Layer-Specific Retinal Perfusion as a Personalized Biomarker: Evaluating the Subclinical Microanatomical Effects of Intracameral Cefuroxime After Routine Cataract Surgery

**DOI:** 10.3390/jpm16060320

**Published:** 2026-06-15

**Authors:** Chia-Yu Wang, Chun-Yao Cheng, Yi-Jie Peng

**Affiliations:** 1 Department of Ophthalmology, Taipei Tzu Chi Hospital, New Taipei City 23142, Taiwan; lucky0helen@gmail.com; 2 Department of Medical Education, National Taiwan University Hospital, Taipei City 100225, Taiwan; b05401143@ntu.edu.tw; 3 Graduate Institute of Clinical Medicine, Chang Gung University, Taoyuan City 33302, Taiwan

**Keywords:** personalized ophthalmology, microvascular biomarkers, macular perfusion, intracameral cefuroxime, phacoemulsification, optical coherence tomography angiography

## Abstract

**Background/Objectives**: The objective of this study was to evaluate macular perfusion changes after intracameral injection (ICI) of cefuroxime at the end of phacoemulsification. **Methods**: Patients who underwent routine phacoemulsification were enrolled. Subjects in the case group had ICI 1 mg/0.1 mL cefuroxime at the end of surgery. Using optical coherence tomography angiography (OCT-A), macular perfusions were assessed at T0 (before surgery), T1, T10, T30, and T90 (days after surgery). Perfusion parameters were calculated in the superficial capillary plexus (SCP) and the deep capillary plexus (DCP). Independent *t*-tests were used to compare the changes from baseline in each parameter between groups. **Results**: A total of 33 eyes in the case group and 27 eyes in the control group were enrolled. After surgery, the case group showed a less pronounced reduction in the foveal avascular zone (FAZ) in the DCP at T10 (−0.06 ± 0.23 vs. −0.18 ± 0.18 mm^2^, *p* = 0.041) and T30 (−0.04 ± 0.20 vs. −0.16 ± 0.24 mm^2^, *p* = 0.050). At T90, there was no statistically significant difference in the FAZ change in the DCP between the groups. The postoperative changes in the vessel density, skeleton density, and acircularity index of the FAZ in the SCP and DCP, central retinal thickness, and best-corrected visual acuity were similar between the groups in all 3 months. **Conclusions**: Our findings indicate that intraoperative ICI low-dose cefuroxime is associated with a temporary deceleration in FAZ reduction in the DCP during the first postoperative month. From a personalized medicine perspective, these layer-specific microanatomic variations suggest that, while prophylactic cefuroxime is globally safe—demonstrating no evidence of inducing capillary dropout, aggravating macular thickening, or compromising visual outcomes within this cohort—preoperative and postoperative OCT-A monitoring can serve as an individualized screening framework to track subclinical perfusion dynamics, especially in patients with compromised retinal baselines.

## 1. Introduction

Modern cataract surgery is a common ophthalmic practice with a high success rate and good visual prognosis. To mitigate the risk of postoperative endophthalmitis (POE), the use of prophylactic intraocular antibiotics has become a widely adopted practice in recent years. Cefuroxime, given at a low-dose concentration (1 mg/0.1 mL) via intracameral injection (ICI), can significantly reduce POE and is widely used in Europe as a standard in cataract surgery procedures [1]. However, complications of acute maculopathy have begun to appear sporadically. In 2010, Buyukyildiz et al. published the first report of serous neurosensory retinal detachment in two patients (2 mg/0.1 mL cefuroxime) [2]. After that, several case reports and observational studies showed extensive macular cystoid changes and serous detachment during the first week postoperatively, which resolved spontaneously in patients who received low-dose cefuroxime [1,2,3,4,5,6]. Especially in vitrectomized cases without vitreous body protection, patients developed a higher incidence of cefuroxime toxic retinopathy (CTR), and even severe visual loss [7,8,9]. Clinical modeling confirms that vitrectomized eyes experience significantly accelerated drug distribution within the posterior segment, and even standard prophylactic doses can rapidly approach toxic thresholds depending on anatomical variations and localized incisional leaks [10]. Unfortunately, in cases that received inadvertent high-dose cefuroxime injection, patients developed permanent macular injury such as foveal avascular zone (FAZ) enlargement, white sclerotic retinal vessels, and hemorrhagic infarction [11,12,13]. These complications underscore that standardized doses can trigger unpredictable macular responses, driven by patient-specific susceptibility and individual barrier integrity.

To the best of our knowledge, previous studies have rarely discussed the effect of cefuroxime on the macula using optical coherence tomography angiography (OCT-A). With the help of OCT-A, separate layers of the retina can be visualized and many perfusion parameters can be easily calculated, which overcomes the limitations of fluorescein angiography (FAG). By avoiding contrast-related risks, OCT-A provides rapid, non-invasive, and highly reproducible layer-specific quantitative analysis, while delivering sharper FAZ border delineation than conventional FAG to capture subtle microanatomic shifts [14]. Preoperative OCT-A images also help to identify subclinical clues of microanatomic changes after surgery. Besides CTR, postoperative cystoid macular edema (CME) can result from another etiology, Irvine–Gass syndrome (IGS), which is the most common cause of unexpected vision loss after uneventful cataract surgery [15]. IGS is usually identified 1–3 months postoperatively and needs to be differentiated from CTR [3]. Therefore, we designed this 3-month follow-up study to compare changes in macular perfusion, thickness, and surgical outcomes between patients with and without ICI low-dose cefuroxime at the end of uneventful cataract surgery. The secondary goal was to compare postoperative macular microanatomies with preoperative self-images within each group to establish individual vascular baselines. Importantly, within the framework of personalized medicine, evaluating these subclinical and layer-specific microanatomical alterations via longitudinal self-comparison allows clinicians to identify individual variations in retinal vascular reactivity. This approach shifts cataract prophylaxis from a universal protocol to a patient-specific risk-stratification model, thereby optimizing patient safety against unexpected retinotoxity.

## 2. Materials and Methods

### 2.1. Participants

We prospectively recruited patients diagnosed with senile cataracts and scheduled for routine phacoemulsification surgery at Taipei Tzu Chi Hospital, New Taipei, Taiwan, from November 2021 to February 2022. This study was conducted in accordance with the Declaration of Helsinki. Approval was obtained from the institutional review board, and each patient provided written informed consent. Given that intracameral antibiotic injection is not universally mandated as the standard routine in Taiwan—where prophylaxis predominantly relies on perioperative povidone–iodine disinfection and postoperative topical antibiotics—the use of a BSS control group was specifically reviewed, approved by the IRB, and disclosed to all participants.

The exclusion criteria included poor preoperative image quality (signal strength index (SSI) < 7/10); prior laser or intraocular surgery or intravitreal treatments (e.g., antivascular endothelial growth factor or corticosteroid); history of maculopathy or uveitis; systemic steroid or antidiuretic use; severe cardiovascular, renal, or liver dysfunction; and surgical complications, including capsular tear and vitreous loss.

### 2.2. Surgical Procedure

All patients were blinded to their group assignments. They received one drop of tropicamide 30 min before surgery, followed by 3 min of eyelid margin disinfection and 1 min of ocular surface disinfection using 10% and 5% povidone–iodine, respectively. A clear corneal incision of 2.2 mm and a paracentesis were placed at the 4 o’clock and 8 o’clock positions, respectively. Routine phacoemulsification procedures were performed with a Centurion^®^ device (Alcon Inc., Fort Worth, TX, USA) for no longer than 15 min. The phacoemulsification energy was delivered in pulse mode. The machine automatically recorded the cumulative dissipated energy (CDE) of ultrasound and the total ultrasound time (TUS). To eliminate surgical duration and energy delivery as confounding variables, all operations were limited to uneventful procedures under 15 min, and the intraoperative CDE metrics were statistically analyzed and balanced between the cohorts. Patients in the cefuroxime group (CEF group) received ICI 0.1 mL of 1 mg/0.1 mL cefuroxime at the end of surgery. In contrast, patients in the control group received ICI 0.1 mL of BSS fluid at the end of surgery. Intraoperative epinephrine injection was not added to the irrigation solution but was administered intracamerally solely in specific cases requiring pupillary dilation (2 eyes in each group; Table 1). All subjects received oral acetazolamide tablets 250 mg twice on the operative day. On postoperative days 1 to 10, the universal topical regimen consisted of levofloxacin 0.5% eye drops (Cravit, Santen, Osaka, Japan) four times daily, tobramycin 0.3%/dexamethasone 0.1% eye drops (Tobradex, Alcon, Fort Worth, TX, USA) four times daily, and a combination eye ointment of 0.1% betamethasone valerate and 0.5% neomycin sulfate (Betason-N, Winston, Taiwan) at bedtime. On postoperative days 11 to 30, the regimen transitioned to sulfamethoxazole 3% eye drops (Sinomine, Sinphar, Taiwan) four times daily and betamethasone 0.1% (Betame, Madison, Taiwan) four times daily. No non-steroidal anti-inflammatory eye drops were routinely administered in this study.

### 2.3. Ocular Examinations

All patients underwent complete ocular examinations, including best-corrected visual acuity (BCVA), intraocular pressure (IOP), slit lamp examination, fundoscopy, spectral domain OCT, and OCT-A, all at T0 (before surgery), T1, T10, T30, and T90 (days after surgery). All exams were performed in the morning. To ensure stable conditions, all patients received at least 30 min of rest at the clinic and underwent a blood pressure recheck before OCT and OCTA image capture.

We used a Zeiss CIRRUS^®^ 6000 (ZEISS, Dublin, CA) to capture 6 × 6 mm macular scans (SSI had to be ≥7/10). With the foveola as the center, the average thickness within the central 1 mm diameter circle was referred to as the central macular thickness (CMT) (ETDRS region 1). The average thickness in the pericentral ring, with a diameter of 1 mm to 3 mm from the center to the outside, was referred to as the inner macular thickness (IMT) (including ETDRS regions 2, 3, 4, and 5). Moreover, the average thickness in the peripheral ring, with a diameter of 3 mm to 6 mm from the center to the outside, was referred to as the outer macular thickness (OMT) (including ETDRS regions 6, 7, 8, and 9). The retinal thickness of the 9 ETDRS regions was obtained automatically by the CIRRUS^®^ macular cube. The superficial capillary plexus (SCP) was in the slab between the internal limiting membrane and the inner plexiform layer (IPL). The deep capillary plexus (DCP) was in the slab between the IPL and the outer plexiform layer.

**Figure 1:**
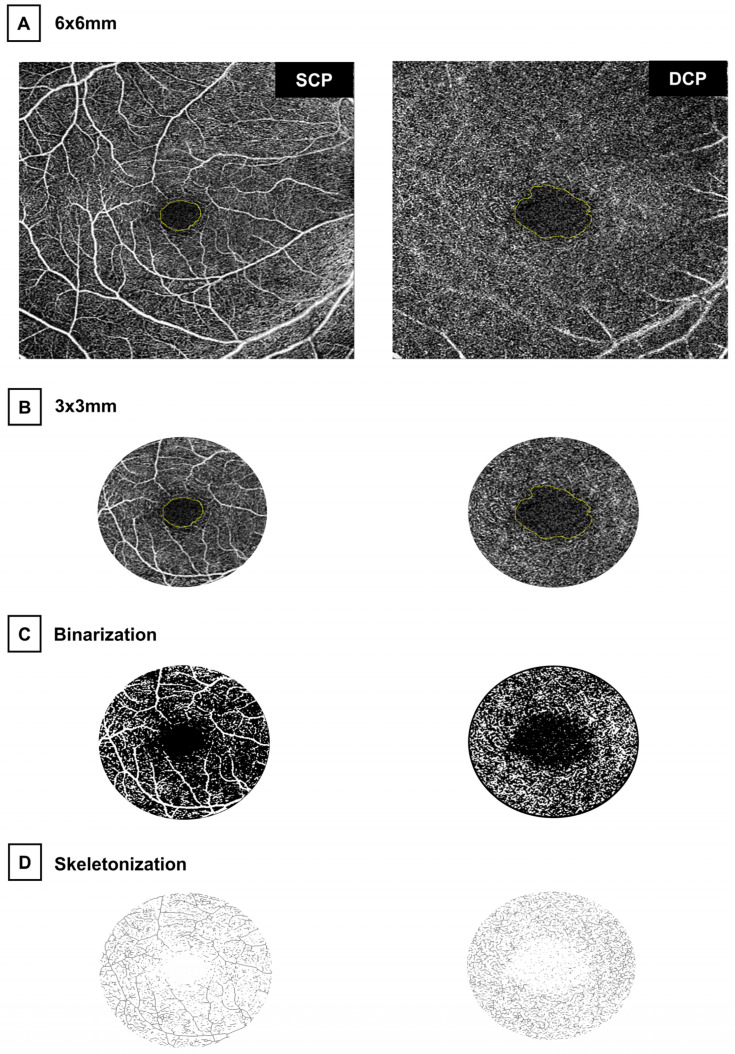
OCT-A image processing. (**A**) Two 6 × 6 mm macula scanning pictures of SCP (**left**) and DCP (**right**) from the same patient, taken with a CIRRUS^®^ 6000. Both were exported into the ImageJ software. Then, the FAZ border in each picture was outlined manually by two ophthalmologists under masking (yellow lines). The mean pixel intensity inside the FAZ area was subtracted to remove noise. (**B**) A 3 mm diameter circle centered on the fovea in each picture. (**C**) Binarization and VD calculated with the software as the ratio of white pixels to the total pixels in the circle. (**D**) Skeletonization and SD calculated with the software as the ratio of black pixels to the total pixels in the circle. Abbreviations: SCP = superficial capillary plexus; DCP = deep capillary plexus; FAZ = foveal avascular zone.

As shown in Figure 1, we exported OCT-A images into ImageJ 1.53 (NIH, USA). To ensure measurement reliability, the tracking boundaries of the FAZ in both the SCP and DCP were independently outlined by two trained graders who were strictly masked to each other’s results and to the clinical group assignments. In alignment with the established ophthalmic literature, this computer-assisted manual delineation demonstrates high reproducibility, yielding an intraclass correlation coefficient (ICC) exceeding 0.90 [16]. After noise reduction, Otsu’s thresholding method was applied within a 3 mm circle centered on the fovea to binarize and skeletonize the images before calculating vessel density (VD) and skeleton density (SD) in each plexus. VD was calculated as the ratio of the total vascular area to the total number of pixels in the selected area, while SD was calculated as the ratio of the total vessel length to the total pixels [17]. Additionally, the acircularity index (AI) of the FAZ was calculated as the ratio of the FAZ perimeter to the perimeter of a circle with an equivalent area; an AI of 1.0 indicates a perfect circle, with increasing values reflecting progressive deviation from a circular shape [18].

### 2.4. Statistical Analysis

The statistical analysis was performed with SPSS^®^ Version 20 (IBM, USA). The normality of continuous variables was verified using the Shapiro–Wilk test and visual inspection of Q-Q plots prior to parametric analysis. Independent t-tests were used to compare the changes in each parameter between the groups at each follow-up visit. A paired *t*-test was used to compare postoperative measurements with preoperative values for perfusion parameters and logMAR VA within each group. A one-way repeated-measures ANOVA was used to analyze the time profile of macular thickness (MT) within each group. *p* < 0.05 was deemed statistically significant.

## 3. Results

### 3.1. Demography

We recruited 88 eyes of 83 Asian individuals (Table 1). Of them, 25 eyes with poor preoperative image quality, 16 with post-laser or intraocular treatments, 5 with macular atrophy, 4 with severe systemic diseases, 3 with thick epiretinal membrane (ERM), and 1 with uveitis were excluded. Finally, 60 eyes of 57 subjects were enrolled in the study: 33 eyes in the case group and 27 eyes in the control group. There was no difference between the cases and controls in demographic features or baseline parameters.

### 3.2. Perfusion Parameters

**Figure 2:**
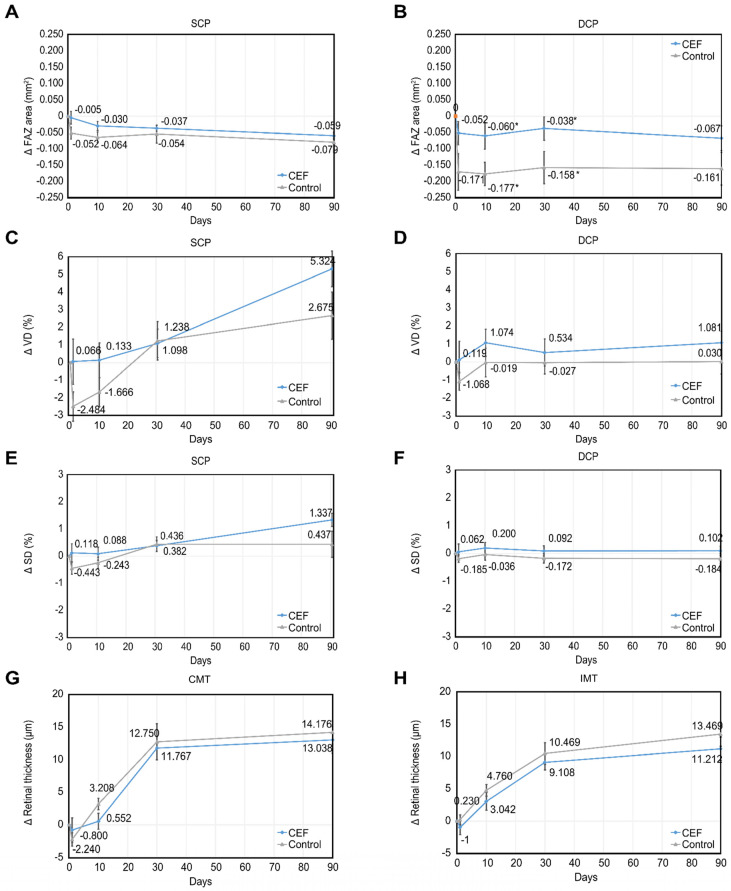
**Changes in macular perfusion and thickness over 3 months after phacoemulsification for each group.** The line graphs show the mean changes in the FAZ area (mm^2^) from baseline values in the (**A**) SCP and (**B**) DCP in the cefuroxime (blue line) and control (gray line) groups. At T10 and T30, there was significantly less reduction in the FAZ area in the DCP in the patients treated with ICI cefuroxime than in those not treated with ICI cefuroxime. The other line graphs show the mean changes in (**C**,**D**) VD (%), (**E**,**F**) SD (%), and (**G**,**H**) macular thickness (μm) over time in each group. There were no statistically significant differences between the two groups in these parameters during the 3-month follow-up. * *p* < 0.05 by independent t-tests. Abbreviations: CEF = cefuroxime group; SCP = superficial capillary plexus; DCP = deep capillary plexus; FAZ = foveal avascular zone; VD = vessel density; SD = skeleton density; CMT = central macular thickness; IMT = inner macular thickness.

As shown in Figure 2 and Online Supplementary Material Table S1, the CEF group had significantly less reduction in the FAZ area from baseline in the DCP than the control group at T10 (−0.06 ± 0.23 vs. −0.18 ± 0.18 mm^2^, *p* = 0.041) and T30 (−0.04 ± 0.20 vs. −0.16 ± 0.24 mm^2^, *p* = 0.050). There was no significant difference in the mean changes in the other OCT-A parameters, including VD, SD, and AI, in the SCP or DCP between the two groups at each follow-up visit. However, there was a trend of higher VD and SD in both the SCP and DCP in the CEF group in all postoperative measurements.

Within-group analysis showed that all postoperative FAZ areas in the DCP were smaller than the preoperative values in the control group (all *p* < 0.05). In the CEF group, all postoperative FAZ areas in the DCP were similar to the preoperative values (all *p* > 0.05). The postoperative FAZ area in the SCP was smaller than the preoperative values at T10, T30, and T90 in the control group (all *p* < 0.05) and at T1, T10, and T90 in the CEF group (all *p* < 0.05). VD and SD in the SCP were both higher than the preoperative values at T90 in the CEF group (VD: 31.34 ± 3.72 vs. 26.01 ± 5.24%, *p* < 0.001; SD: 7.72 ± 0.95 vs. 6.38 ± 1.27%, *p* < 0.001). The other patterns of the perfusion parameters were unremarkable.

### 3.3. Macular Thickness

As shown in Figure 2 and Online Supplementary Material Table S1, the two groups had similar results for the mean change in CMT and IMT at each follow-up visit. Within-group analysis showed that both groups had a thicker CMT and IMT at T30 and T90 than at earlier follow-up visits (all *p* < 0.05) (Table 2).

### 3.4. Visual Outcomes and Complications

The mean change in BCVA was similar between the two groups at each follow-up visit (online Supplementary Material Table S1). Within-group analysis of BCVA showed that all participants had significant improvements after surgery (all *p* < 0.05), except T1 vision in the CEF group (*p* = 0.05). No participant had elevated IOP above 25 mmHg during the study. No CME or POE was diagnosed.

## 4. Discussion

To the best of our knowledge, this is the first prospective observational study with a single-blind subject test to investigate changes in macular circulation and thickness after ICI cefuroxime at the end of routine cataract surgery, accompanied by OCT-A images, preoperative self-data, and a longer follow-up duration.

### 4.1. Macular Perfusion

This initial section establishes the baseline tracking of FAZ dimensions following routine cataract removal, examining the natural microvascular adaptations and potential optical artifacts that occur independently of antibiotic influence. OCT-A is a high-resolution, non-invasive technique that efficiently assesses the retinal microanatomy, especially by calculating FAZ parameters that are not affected by dye leakage on the FAG. Moreover, FAZ measurements are the most robust parameters, as lens opacities generally do not obscure their delineation [19]. In this study, we demonstrated that the postoperative FAZ areas in both the SCP and DCP were significantly smaller than their baseline values in control eyes without ICI cefuroxime. This finding aligns with previous studies assessing the FAZ in normal eyes undergoing uneventful phacoemulsification surgery. Crucially, the surgical removal of a cataract enhances optical media clarity and minimizes signal attenuation, which can significantly alter the automated boundary detection on OCT-A, thereby resulting in an apparent reduction in the measured FAZ area. For instance, Yang et al. (34 eyes) and Zhao et al. (32 eyes) found significant postoperative FAZ reduction in the SCP (*p* < 0.001) and DCP (*p* = 0.003) that persisted up to T30 and T90, respectively [20,21]. Similarly, Yu et al. (11 normal eyes) and Antonio et al. (14 eyes with less severe cataracts and nine eyes with severe cataracts) found a comparable trend of FAZ reduction in the SCP after surgery [19,22]; however, limited sample sizes might have precluded statistical significance. Conversely, Xinyu et al. described a cohort of 107 normal Chinese eyes that demonstrated a stable postoperative FAZ area; however, because their quantification method failed to differentiate between the SCP and the DCP, the layer-specific microanatomic alterations within each plexus were likely obscured [23]. Based on our findings, we suggest that in patients with preserved retinal vascular elasticity, true physiological recovery paired with post-cataract optical magnification changes collectively manifests as a smaller measured FAZ area in both the SCP and DCP following uneventful cataract surgery.

By evaluating the specific pharmacological impact of cefuroxime, we examined layer-specific FAZ changes and developed a cellular hypothesis involving transient Müller cell stress. Interestingly, for the eyes treated with ICI cefuroxime, we found that although the FAZ area in the DCP showed a reduction trend postoperatively, there was no statistically significant difference from the preoperative size. During the first month after surgery, the FAZ area in the DCP showed a trend toward less reduction than that in the control eyes (although with marginal significance at T10; *p* = 0.041). Based on previously published evidence, CTR is characterized by cystoid changes in the outer retinal layer. It occurs within 24 h postoperatively, persists for 1 week, and then regresses spontaneously. Most cases were managed with supportive care and achieved good visual outcomes. However, severe cases can exhibit extensive serous neurosensory retinal detachment, disruption of the photoreceptor ellipsoid zone, and global retinal dysfunction on a full-field electroretinogram (ERG), with a guarded visual outcome [12,24]. The pathophysiology of CTR remains unclear. Zuo et al. suggested that it was probably related to transient failure of the retinal pigment epithelium (RPE) sodium–potassium pump [3]. Faure et al. observed that cefuroxime was toxic to Müller cell function, showing sunburst-pattern CME due to the Müller cell anatomy in Henle’s fiber layer [24]. In a rabbit model, Shahar et al. found permanent ERG deficits and histologic structural damage in the retina in those treated with ICI high-dose cefuroxime (10 mg/0.1 mL), but not in those treated with ICI low-dose cefuroxime (1 mg/0.1 mL). Interestingly, they further used glial fibrillary acidic protein (GFAP)—an intermediate filament that is normally not expressed by Müller cells, though in stress situations, such as retinal ischemia and retinal detachment, it will be expressed in these cells—to stain rabbit retinas and found GFAP immunoreactivity in Müller cells in both dose groups [25]. This means that low-dose cefuroxime probably induced sufficient retinal stress to stimulate Müller cell activity. Müller cells play a role in controlling retina biomechanics. They are the primary structural support for the foveola, acting as a plug that binds cells together [26]. By up- or downregulating their intermediate filaments, such as GFAP and vimentin, they can alter the entire retina tissue [27,28]. In addition, retinal capillaries are ensheathed by the foot processes of Müller cells as part of the structural organization of the blood–retina barrier (BRB) [28,29]. Upon surgical trauma or chemical irritation, these cells can transition into a reactive phenotype, triggering reactive gliosis and releasing specific metabolic cascades [28].

Based on these insights, we hypothesize that the less pronounced FAZ reduction in the cefuroxime-treated eyes might be linked to a transient and subclinical Müller cell dysfunction rather than irreversible structural damage. Consequently, these eyes displayed a delayed pattern of FAZ reduction compared to the control eyes. Since the SCP is connected proximally to the retinal arterioles, it has a greater perfusion pressure and is inherently less vulnerable than the DCP [30]. This anatomically driven difference may explain why the subtle FAZ alterations were exclusively observed in the DCP within this cohort. Another potential contributing factor is that the DCP possesses a greater baseline FAZ area, making its minor fluctuations more statistically detectable. This layer-specific vulnerability aligns with in vivo murine models, demonstrating that intravitreal cefuroxime toxicity extends into the inner nuclear layer [31]. Because the DCP sits right at the boundary of this injured layer, this tissue stress directly impacts the deep capillaries [31].

Furthermore, large-scale database modeling demonstrates that intraocular cefuroxime dilution kinetics are strictly governed by an individual’s ocular volume and axial length, placing hypermetropic small eyes at an inherently higher risk of drug accumulation and localized toxicity [10]. Similarly, vitrectomized eyes face a higher risk of toxicity because they lack vitreous protection, leading to accelerated drug distribution in the posterior segment [10]. In the era of personalized medicine, pre-surgical screening must therefore look beyond blood vessel health to include physical eye dimensions and surgical history. Combining layer-specific OCT-A tracking with eye-volume measurements allows clinicians to preemptively identify these highly vulnerable patients, shifting cataract management toward a personalized approach that effectively prevents patient-specific complications. While these pathophysiological explanations remain speculative due to the lack of direct cellular and molecular evidence in this clinical trial, they offer a biologically plausible framework that warrants further experimental validation.

Beyond the avascular zone dynamics, the evaluation was expanded to peripheral perfusion metrics—namely, vascular density (VD) and skeletal density (SD)—to comprehensively assess capillary preservation and vasodilation. VD, FAZ size, and FAZ shape are used to investigate capillary dropout in retinopathy. Large vessels play a more important role than capillaries in affecting VD, while large vessels and capillaries contribute equally to the SD calculation [16,32]. In this study, during the 3-month postoperative period, the eyes in the case group displayed a tendency towards higher VD and SD in both the SCP and DCP compared to the control group (Figure 2C,D). Interestingly, at T90, the eyes in the case group exhibited significantly increased VD and SD in the SCP compared to their baseline values. These results suggested that ICI cefuroxime caused vasodilation in both large retinal vessels and small capillaries and achieved its maximum effect at T90. The neurovascular coupling effect may have stimulated Müller cells to release the vasoactive metabolites of arachidonic acid, contributing to vasodilation [33]. Moreover, previous studies showed no active dye leakage on FAG in patients with CTR [24,34,35]. This means that the vascular change phenomenon caused by ICI cefuroxime would not disrupt the BRB. Reassuringly, low-dose cefuroxime administration showed no evidence of causing capillary dropout in our study. This conclusion is supported by the unchanged AI of the FAZ in all eyes before and after surgery, indicating no alteration in vessel distribution or FAZ irregularity.

### 4.2. Macular Thickness

Shifting the focus from microvascular perfusion to retinal structural morphology, this subsection delineates the clinical dynamics of postoperative macular thickness (MT) variations and the potential influence of perioperative medications. In this study, the eyes treated with cefuroxime did not exhibit a larger increase in MT than the control eyes in any of the 3 months. Irvine first described IGS after intracapsular cataract extraction in 1953 [15]. The incidence of clinically significant IGS impairing patients’ vision has decreased to 1–2% among those who receive small-incision phacoemulsification [15,36]. Currently, based on OCT diagnosis, IGS-associated CME with intraretinal cysts occurs in 5% of patients, while macular thickening without cysts occurs in 7% [37]. For eyes without cysts, macular thickening is unlikely to disturb the surgical visual outcome. For example, MT increased in the first 3 months and returned to baseline at 6 months postoperatively, as reported by Muhammed et al. [38]. There was no correlation between MT and BCVA in the study by Jagow et al. [39]. While we excluded advanced IGS risk factors (such as active uveitis or tractional ERM), our cohort still included eyes with mild-to-moderate NPDR or non-tractional ERM. Retaining these subtle baselines is highly relevant; large-scale real-world data from the Intelligent Research in Sight (IRIS^®^) Registry involving 3.1 million eyes demonstrates that preexisting diabetic retinopathy and ERM compound the risk of postoperative macular complications by 8.20-fold and 4.74-fold, respectively [40]. Monitoring these subclinical presentations provides valuable insight into how standard prophylactic doses of cefuroxime interact with compromised macular tissues. After phacoemulsification, regardless of whether the eyes were treated with or without ICI cefuroxime, there was an increase in macular thickness, but no cystoid change was observed. The degree of MT rose steadily in the first month and then more slowly at 2–3 months postoperatively, forming a similar trajectory in both groups. As in other studies, the patients maintained good visual acuity after surgery.

To ensure scientific rigor, potential confounding variables that independently influence macular thickness—including surgical energy metrics and the identical postoperative medication regimen—must be evaluated. In this trial, the ultrasonic parameters—specifically CDE (*p* = 0.56) and TUS (*p* = 0.59)—were symmetrically distributed without statistical discrepancies between the groups, ensuring that the delivered intraocular acoustic energy did not act as a confounding factor. Nevertheless, the microstructural impact of this surgical energy warrants consideration. Recent clinical evidence demonstrates that higher intraoperative ultrasound energy expended during phacoemulsification correlates significantly with subclinical thinning and tissue stress within the deeper retinal layers postoperatively, whereas the inner retinal layers exhibit diffuse inflammatory thickening [41]. This layer-specific architectural response shows that surgical energy independent of pharmacological interventions can directly modulate localized retinal thickness trajectories. Because our CDE and TUS metrics were uniformly distributed, this baseline acoustic stress affected both groups symmetrically, confirming that the observed post-cataract variations reflect genuine pharmacological trends rather than underlying surgical discrepancies. Additionally, all participants universally received oral acetazolamide (250 mg BID) on the operative day and an identical two-phase postoperative topical anti-inflammatory regimen (consisting of combined steroid–antibiotic agents followed by maintenance corticosteroid drops). While carbonic anhydrase inhibitors and topical steroids are known to alter macular thickness dynamics by facilitating fluid clearance [15,42], their universal application across both cohorts eliminated any potential confounding bias during inter-group comparisons. Consequently, the observed temporary deceleration in DCP FAZ reduction in the CEF group reflects a genuine pharmacologically induced trend rather than baseline surgical or medical discrepancies.

Integrating these microvascular and structural insights into personalized clinical workflows offers significant therapeutic value. While low-dose ICI cefuroxime demonstrated an excellent overall safety profile, the transient FAZ deceleration observed exclusively in the DCP underscores the utility of pre-surgical risk screening. In the era of personalized medicine, leveraging layer-specific OCT-A metrics alongside structural OCT imaging to map out an individual’s microscopic tolerance enables ophthalmologists to identify vulnerable retinal baselines—such as preexisting subclinical microangiopathies or vitreoretinal interface disorders—thereby allowing for tailored perioperative protocols or alternative endophthalmitis prophylaxis.

### 4.3. Study Limitations and Future Directions

Firstly, this study is limited by its relatively small sample size (33 eyes vs. 27 eyes) and mild baseline heterogeneity. Although patients with advanced ocular pathologies were strictly excluded, the inclusion of a limited number of cases with mild to moderate NPDR, mild ERM, and controlled POAG introduces potential confounding factors that could independently affect retinal microvasculature and OCT-A parameters. While these comorbidities were limited and comparably distributed between the CEF and control groups, their potential influence could not be entirely eliminated within this compact cohort. Secondly, we acknowledge a potential optical media clarity artifact: the removal of cataracts naturally enhances postoperative OCT-A signal strength and quality, which can mathematically shift FAZ boundary definitions. Although this signal enhancement occurred symmetrically in both groups due to identical surgical procedures, it remains a potential source of measurement variability. Thirdly, due to the clinical challenges of definitively diagnosing complete or anomalous posterior vitreous detachment preoperatively in cataractous eyes, the precise vitreous status was not fully documented. Although we strictly excluded cases with active vitreomacular traction or thick ERM to protect foveal anatomy, subtle subclinical variations in the vitreomacular interface could not be entirely ruled out. Furthermore, a post hoc power analysis based on the primary endpoint (FAZ area change in the DCP at T10) revealed a Cohen’s *d* of 0.58 and an achieved statistical power of 60.0%. Consequently, some of our statistically significant findings showed marginal *p*-values, and our results should be interpreted as trend-indicating rather than definitive conclusions. Although the inter-group difference in the DCP FAZ reduction diminished by the third postoperative month, future large-scale, multicenter, randomized controlled trials with stricter cohort homogeneity, extended follow-up durations, and objective electrophysiological evidence are warranted to validate these trends. Overall, our study elucidated the outlook of macular microvascular changes following ICI CEF in healthy patients after cataract surgeries and may serve as a preliminary survey for further personalized utility of ICI cefuroxime in complicated cases with vitreoretinal interface pathologies or macular microangiopathies. Lastly, clarifying the exact subcellular mechanisms underlying potential cefuroxime-induced retinal stress remains a critical direction for future research.

## 5. Conclusions

In conclusion, although the safety of prophylactic ICI low-dose cefuroxime at the end of cataract surgery is widely accepted, it may induce mild, layer-specific macular perfusion changes characterized by a temporary deceleration in FAZ reduction in the DCP. From a personalized medicine perspective, these microvascular variations suggest that, while the intervention remains globally safe—demonstrating no negative impact on vascular distribution, aggravation of macular thickening, or disturbance of surgical outcomes within this cohort—preoperative and postoperative OCT-A monitoring can serve as an individualized screening framework to track subclinical perfusion dynamics, especially in patients with compromised retinal baselines. Further large-scale, randomized prospective investigations are warranted to confirm these individualized trends.

## Figures and Tables

**Table 1:** Subject demography.

	CEF Group	Control Group	*p* Value ^1^
	33 eyes of 31 patients	27 eyes of 26 patients	
Sex			
Male	8	8	
Female	23	18	
Age (years)	68.6 ± 10.2	66.0 ± 9.3	0.31
CDE (percent-seconds)	13.6 ± 4.6	14.6 ± 6.8	0.56
TUS (seconds)	80.45 ± 21.32	84.12 ± 28.05	0.59
Intraoperative epinephrine injection (eyes)	2	2	
Ocular problems (eyes)			
Mild NPDR	0	2	
Mild to moderate NPDR	1	1	
Mild ERM	4	5	
POAG	1	2	

^1^ Independent *t*-tests. Abbreviations: CEF = cefuroxime; CDE = intraoperative cumulative dissipated energy of ultrasound; TUS = total ultrasound time; NPDR = nonproliferative diabetic retinopathy; ERM = epiretinal membrane; POAG = primary open angle glaucoma.

**Table 2:** The difference in macular thickness between baseline and various follow-up visits after surgery.

**CMT (μm) ** ** (** ***p*** ** < 0.001 ^1^** **)**					**IMT (μm) ** ** (** ***p*** ** < 0.001 ^1^** **)**				
Control	T1	T10	T30	T90	Control	T1	T10	T30	T90
T0	−2.79	2.21	9.00 *	12.57 *	T0	0.52	3.91 *	8.46 *	12.50 *
T1		5.00 *	11.79 *	15.36 *	T1		3.39 *	7.95 *	11.98 *
T10			6.79 *	10.36 *	T10			4.55 *	8.60 *
T30				3.57	T30				4.04
T90					T90				
CMT (μm) (*p* < 0.001 ^1 ^)					IMT (μm) (*p* < 0.001 ^1 ^)				
CEF	T1	T10	T30	T90	CEF	T1	T10	T30	T90
T0	0.36	1.14	11.28 *	12.68 *	T0	−0.92	3.11	8.10 *	10.15 *
T1		0.73	10.86 *	12.32 *	T1		4.03 *	9.02 *	11.08 *
T10			10.09 *	11.55 *	T10			4.99 *	7.04 *
T30				1.46	T30				2.05
T90					T90				

* *p* < 0.05 analyzed by multiple comparisons between various follow-up visits for macular thickness. ^1^ One-way repeated ANOVA. Abbreviations: CMT = central macular thickness; IMT = inner macular thickness.

## Data Availability

The original contributions presented in this study are included in this article/the Supplementary Materials. Further inquiries can be directed to the corresponding author.

## Supplementary Materials

The following supporting information can be downloaded at https://www.mdpi.com/article/10.3390/jpm16060320/s1: Table S1: Comparisons between the cefuroxime (CEF) and control groups before and after surgery.

## Abbreviations

The following abbreviations are used in this manuscript:

ICI	Intracameral Injection
OCT-A	Optical Coherence Tomography Angiography
SCP	Superficial Capillary Plexus
DCP	Deep Capillary Plexus
FAZ	Foveal Avascular Zone
POE	Postoperative Endophthalmitis
CTR	Cefuroxime Toxic Retinopathy
FAG	Fluorescein Angiography
CME	Cystoid Macular Edema
IGS	Irvine–Gass Syndrome
SSI	Signal Strength Index
CDE	Cumulative Dissipated Energy
TUS	Total Ultrasound Time
CEF	Cefuroxime Group
BCVA	Best-corrected Visual Acuity
IOP	Intraocular Pressure
MT	Macular Thickness
CMT	Central Macular Thickness
IMT	Inner Macular Thickness
OMT	Outer Macular Thickness
ICC	Intraclass Correlation Coefficient
VD	Vessel Density
SD	Skeleton Density
AI	Acircularity Index of the FAZ
ERM	Epiretinal Membrane
NPDR	Nonproliferative Diabetic Retinopathy
POAG	Primary Open Angle Glaucoma
ERG	Electroretinogram
RPE	Retinal Pigment Epithelium
GFAP	Glial Fibrillary Acidic Protein
BRB	Blood–Retina Barrier
